# 4-Аminopyridine sequesters intracellular Ca^2+^ which triggers exocytosis in excitable and non-excitable cells

**DOI:** 10.1038/srep34749

**Published:** 2016-10-05

**Authors:** Ludmila A. Kasatkina

**Affiliations:** 1 The Department of Neurochemistry, Palladin Institute of Biochemistry, NAS of Ukraine 9, Leontovicha Street, Kyiv, 01030, Ukraine

## Abstract

4-aminopyridine is commonly used to stimulate neurotransmitter release resulting from sustained plasma membrane depolarization and Ca^2+^-influx from the extracellular space. This paper elucidated unconventional mechanism of 4-aminopyridine-stimulated glutamate release from neurons and non-neuronal cells which proceeds in the absence of external Ca^2+^. In brain nerve terminals, primary neurons and platelets 4-aminopyridine induced the exocytotic release of glutamate that was independent of external Ca^2+^ and was triggered by the sequestration of Ca^2+^ from intracellular stores. The initial level of 4-aminopyridine-stimulated glutamate release from neurons in the absence or presence of external Ca^2+^ was subequal and the difference was predominantly associated with subsequent tonic release of glutamate in Ca^2+^-supplemented medium. The increase in [Ca^2+^]_*i*_ and the secretion of glutamate stimulated by 4-aminopyridine in Ca^2+^-free conditions have resulted from Ca^2+^ efflux from endoplasmic reticulum and were abolished by intracellular free Ca^2+^ chelator BAPTA. This suggests that Ca^2+^ sequestration plays a profound role in the 4-aminopyridine-mediated stimulation of excitable and non-excitable cells. 4-Aminopyridine combines the properties of depolarizing agent with the ability to sequester intracellular Ca^2+^. The study unmasks additional mechanism of action of 4-aminopyridine, an active substance of drugs for treatment of multiple sclerosis and conditions related to reduced Ca^2+^ efflux from intracellular stores.

The intercoupling of biologically active substances with the unintended targets is one of the important issues for their pharmacological and research application regardless to the achieving of the desired effect. This in turn can lead to incorrect interpretation of the results in experimental research and become a likely cause of unpredictable side-effects for medical practice. This study has been focused on the action of 4-aminopyridine (4-AP), well known blocker of voltage-activated K^+^-channels, on calcium homeostasis and glutamate transport in excitable and non-excitable cells.

Besides the prevention of presynaptic membrane repolarization and increasing the propagation of action potential along axons, 4-AP can promote Ca^2+^ influx through potential-sensitive Ca^2+^-channels^1^,^2^. By increasing the impulse conduction through demyelinated axons and the neurotransmitter release at the neuromuscular junction, 4-AP reduces the main symptoms of multiple sclerosis. It should be noted that 4-AP still remains the most appreciable within the variety of drugs available for the treatment of this disorder. However, there is growing evidence suggesting the existence of additional mechanisms underlying the 4-AP action on neurotransmission and secretion. For instance, in isolated brain nerve terminals (synaptosomes) 4-AP was shown to stimulate the release of several neurotransmitters with putative dependence on external Ca^2+^. The stimulation of gamma-aminobutyric acid (GABA) and acetylcholine release was dependent on the presence of external Ca^2+^, whereas no clear Ca^2+^-dependence was observed for glutamate release^3^. As an allosteric modulator of pore-forming P2X7 receptor 4-AP is involved in the sustained calcium entry in human mononuclear cells^4^. Moreover, 4-AP is also able to directly stimulate high voltage-activated Ca^2+^-channels^5^ and compete with both agonists and antagonists to α_2_-adrenoreceptors^6^.

This supports the view that the mode by which 4-AP stimulates secretion in different cell types is more complex and, yet, not fully uncovered. In light of the diverse mechanisms of 4-AP action further research is required to discriminate between neurotransmitter release stimulated in the absence and presence of external Ca^2+^. The aim of presented study was to study the origin and mechanism of glutamate release stimulated by 4-AP in excitable and non-excitable cells in the absence of external Ca^2+^. The comparative analysis of the data obtained revealed new properties of 4-AP action common for all types of cells tested.

## Results

### 4-Aminopyridine induced the exocytosis but did not interfere with the proton electrochemical gradient and storage capacity of synaptic vesicles

**Figure 1:**
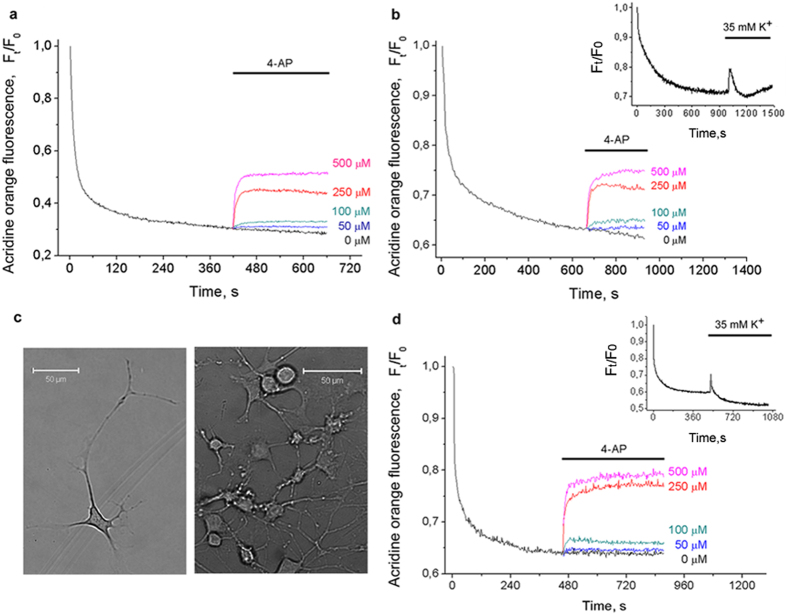
The accumulation of pH-sensitive dye, acridine orange (AO) and its release upon stimulation with 4-AP in the absence of external Ca^2+^: traces for AO dynamics in suspension of platelets (**a**), isolated brain nerve terminals (**b**) and primary neurons (**d**). 4-AP was applied for the duration indicated by the horizontal bars; (**c**) Light microscopic images of primary culture of mouse cortical neurons: single cell (left panel) and neurons in culture (right panel). Scale bar is 50 μm. Insets of (**b**,**d**) demonstrate active exo-/endocytosis in the same preparations of synaptosomes and primary neurons, respectively. Final concentration of total protein was 0.3 mg/mL. Traces are typical for 10 independent experiments.

Experiments with 4-AP stimulation were performed on blood platelets and on differentiated primary neurons that displayed axonal and dendritic outgrowth and formed the network of cells with neurite branching at 14^th^ day of culturing (Fig. 1c). Isolated brain nerve terminals and primary cultured neurons exhibited a pronounced exocytotic response to high-K^+^ depolarization and subsequent endocytosis in Ca^2+^-supplemented medium (insets of Fig. 1b,d).

**Figure 2:**
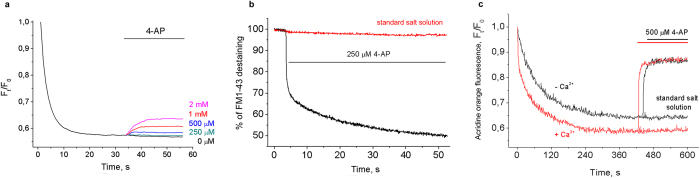
4-AP-stimulated depolarization and exocytotic release of pH-sensitive dye AO. (**a)** Dose-dependent depolarization of membrane potential stimulated by 4-AP in preparation of cortical neurons. Recordings were made using potential-sensitive dye rhodamine 6G with excitation and emission wavelengths of 528 and 551 nm, respectively. The curves were normalized to those in the absence of cells; (**b)** Time course of FM1-43-loaded synaptic vesicle destaining during 4-AP stimulation of primary neurons. Red trace indicates the fluorescence intensity profile in standard salt solution without stimulation. Destaining was calculated in percentage of total accumulated dye; (**c**) The comparative analysis of 4-AP-stimulated AO release from cortical synaptosomes with and without external Ca^2+^ (2 mM). Recordings were made at excitation and emission wavelengths of 490 and 530 nm, respectively. The curves were normalized to those in the absence of synaptosomes. The 4-AP was applied for the duration indicated by the horizontal bar. Final concentration of total protein was 0.3 mg/mL. Traces were typical for 7 independent experiments.

At first, 4-AP was found to decrease the acidification of intracellular compartments in platelets, isolated brain nerve terminals and neurons (Fig. 1a,b,d). Spectrofluorimetric registration with pH-sensitive fluorescent dye acridine orange (AO), which is typically used for monitoring the changes in ΔpH across the intracellular acidic compartments, demonstrated that 4-AP is able to dissipate the ΔpH or stimulate the exocytotic release. To test if this effect is dose-dependent cells were stimulated with different concentrations of 4-AP (50–500 μM). It is worth of noticing that 4-AP is able to produce this effect at lower concentrations (50–250 μM) than required to depolarize plasma membrane (1-2 mM, Fig. 2a).

To verify if 4-AP is able to stimulate exocytosis the staining with FM1-43 dye was applied. This approach allowed the registration of exocytotic release of vesicle constituents at the last step of SNARE-mediated membrane fusion. The destaining of FM1-43-labelled synaptic vesicles has been demonstrated upon 4-AP application at Ca^2+^-free conditions (Fig. 2b). Stable initial fluorescence intensity and traces registered in standard salt solution indicated the low rate of spontaneous exocytosis and the absence of unspecific dye leakage. The superimposition of FM1-43 destaining traces with the pH-sensitive dye release (Figs 1 and 2b) and neurotransmitter release allowed tracking of membrane fusion with simultaneous release of synaptic vesicle constituents and indicated that 4-AP-stimulated release of AO in Ca^2+^-free medium occurred via exocytosis.

The next step was to compare the 4-AP-stimulated secretion of synaptic vesicle constituents in the absence and presence of external Ca^2+^. The analysis of dye redistribution in Ca^2+^-free and Ca^2+^-supplemented medium demonstrated that 4-AP-induced release of synaptic vesicle constituents from isolated brain nerve terminals is predominately Ca^2+^-independent (Fig. 2c). The accumulation of AO in synaptosomes in the presence of Ca^2+^ (2 mM) increased by 6% compared to Ca^2+^-free conditions. The same difference was inherent for subsequent release of the dye: nerve terminals released 64.8 ± 7.4% and 69.7 ± 6.7% of accumulated dye in the absence of Ca^2+^ and in the presence of 2 mM Ca^2+^, respectively. This property of 4-AP was also demonstrated in the experiments with neurons: Ca^2+^-dependent release was 12% higher than Ca^2+^-independent. The difference in steady-state level of dye uptake in Fig. 2c indicates that additional pool of synaptic vesicles is involved in accumulation and release of AO in the presence of Ca^2+^.

To exclude the unspecific effects of 4-AP due to its base properties or possible interaction with the dye additional experiments were carried out for the acidification and neurotransmitter storage in isolated synaptic vesicles. Synaptic vesicles were loaded with the pH-sensitive dye acridine orange that tends to form dimers and multimers after protonation at acidic pH. This led to the decrease in green fluorescence that is inherent for monomeric dye.

**Figure 3:**
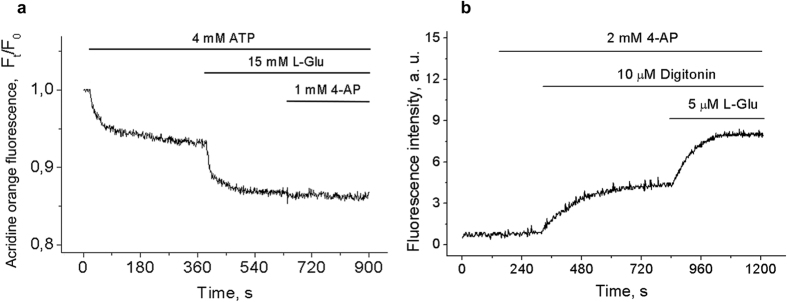
Acidification of isolated synaptic vesicles and retention of endogenous glutamate during application of 4-AP. (**a)** The changes of proton gradient of isolated synaptic vesicles. The quenching of AO green fluorescence represented the generation of inside-acidic ΔpH. Isolated synaptic vesicles were preloaded with AO in the presence of 4 mM ATP. L-glutamate (15 mM) and 4-AP (1 mM) were applied at the steady-state of dye fluorescence for the duration indicated by the horizontal bars. Final concentration of total protein was 0.15 mg/mL; (**b)** The registration of glutamate release from isolated synaptic vesicles using the glutamate dehydrogenase assay. Digitonin (10 μM) was applied to deplete the vesicular glutamate. Final concentration of total protein was 0.5 mg/mL. Traces are typical for 5 independent experiments.

L-glutamate induced additional acidification of synaptic vesicles (Fig. 3a), which is consistent with the current conception of glutamate transport that involves vesicular glutamate transporters VGLUTs^7^. 4-AP was applied once the steady-state level of fluorescence intensity had been reached. The agent failed to induce any noticeable release of pH-sensitive dye from isolated synaptic vesicles at concentrations of 1-2 mM (Fig. 3a). As shown in Fig. 3b, 4-AP did not stimulate the release of endogenous glutamate from isolated synaptic vesicles. These data confirm that 4-AP does not interfere with the proton electrochemical gradient and storage capacity of synaptic vesicles. Thus, 4-AP or signal it generates at intermediate steps of pathway requires the target on plasma membrane, the other intracellular compartment or cytosol.

### Endogenous glutamate release stimulated by 4-AP in Ca^2+^-free conditions

Neurons and platelets have cytosolic and secretable pools of glutamate. The former depends on the functioning of high-affinity glutamate transporters (excitatory amino acid transporters, EAATs) on plasma membrane, vesicular glutamate transport and the activity of enzymes that metabolize glutamate. The secretable pool of glutamate represented by synaptic vesicles in neurons and dense secretory granules in platelets is replenished by vesicular glutamate transporters VGLUT1/2^8^,^9^,^10^.

To estimate the release of endogenous glutamate that follows 4-AP stimulation the glutamate dehydrogenase assay was used. For quantitative analysis the activity of glutamate dehydrogenase was calibrated with the different concentrations of exogenous glutamate at the end of experiments.

**Figure 4:**
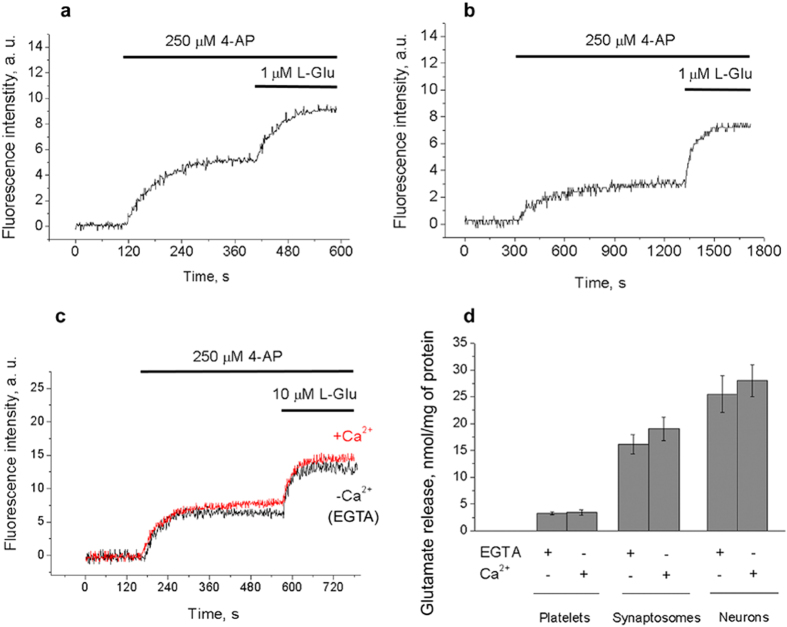
The release of endogenous glutamate. Platelets (**a**), isolated brain nerve terminals (**b**) and neurons (**c**) were stimulated by 4-AP in the absence of external Ca^2+^ and the release of endogenous glutamate was monitored by the changes in fluorescence of NADH; (**d)** The quantitative analysis of 4-AP-stimulated release of endogenous glutamate in the absence or presence of Ca^2+^ (2 mM). Endogenous glutamate release was registered using the glutamate dehydrogenase assay. Final concentration of total platelet/neuronal protein was 0.5 mg/mL. Traces are typical for 10 independent experiments.

Although in some experiments 4-AP was able to provoke glutamate release at concentration of 30 μM the typical and reproducible responses were obtained at 250 μM. Figure 4a shows platelet responses following stimulation in Ca^2+^-free medium: after application of 250 μM 4-AP platelets released 3.45 ± 0.5 nmol of L-glutamate per mg of total platelet protein. The initial level of 4-AP-stimulated glutamate release from neurons and isolated brain nerve terminals in the absence or presence of Ca^2+^ was subequal, and the difference was predominantly associated with the following tonic release of glutamate in Ca^2+^-supplemented medium. In isolated brain nerve terminals and primary neurons in Ca^2+^-free medium 250 μM 4-AP induced the release of 16.2 ± 1.8 nmol and 25.5 ± 3.4 nmol of endogenous glutamate per mg of total synaptosomal/neuronal protein (at 5^th^ min), respectively (Fig. 4b–d). After stimulation with 4-AP in Ca^2+^-supplemented medium isolated brain nerve terminals and neurons released 19.0 ± 2.2 nmol and 28.0 ± 2.9 nmol of endogenous glutamate per mg of protein, respectively (Fig. 4d). Thus, the substantial 4-AP-stimulated release of endogenous glutamate from platelets, isolated brain nerve terminals and neurons was consistent with 4-AP action on the acidification of intracellular compartments (Fig. 1) and represented the process that does not necessarily require external Ca^2+^.

### Surface exposure of dense granule marker CD63 stimulated by 4-AP

In platelets stimulated with ADP, thrombin or other platelet agonist the increase of [Ca^2+^]_*i*_ is a key trigger for massive platelet deformation, secretion of granular constituents, adhesion and spreading. The small volume of interplatelet cleft allows creating high concentration of effectors released from secretory granules even if their total content is low. To detect the secretion in activated platelets the surface exposure of secretory granule markers was studied. During the SNARE-mediated fusion platelet secretory granules may fuse directly with the surface-connected open canalicular system^11^, or merge with each other (compound fusion), and then with the open canalicular system^12^. This mode of secretion is similar to homo- and heterotypic fusion of synaptic vesicles in neurons and results in translocation of membrane-bound proteins of platelet secretory granules on the plasma membrane. CD63 is predominantly associated with intracellular compartments such as dense secretory granules and lysosomes and, therefore, can be employed to detect platelet activation and granule secretion.

**Figure 5:**
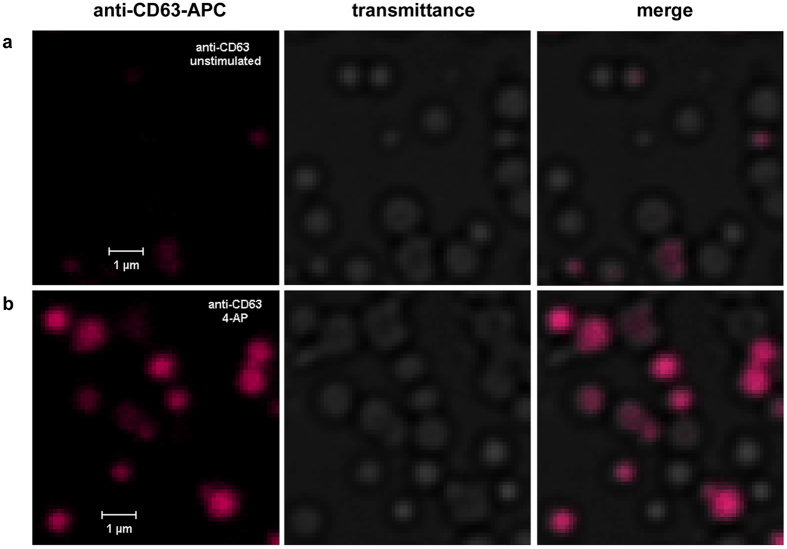
Surface exposure of dense granule marker CD63 in platelet stimulated with 4-aminopyridine. Platelets were stimulated by 4-AP (250 μM) under Ca^2+^-free conditions. Resting (**a**) and 4-AP-stimulated (**b**) platelets were labelled with allophycocyanin-conjugated anti-CD63-mAs for 20 min on ice. Specimens were excited with 633-nm laser and the emission was collected through a 650-nm long pass filter. Transmitted light panel demonstrates the distribution of platelets in specimen. Scale bar is 1 μm.

Laser scanning confocal imaging of platelets labelled with allophycocyanin-conjugated anti-CD63-mAbs in combination with transmitted light imaging demonstrated that under Ca^2+^-free conditions 4-AP application (250 μM) enhanced the surface exposure of CD63 (Fig. 5). The comparative analysis of the fluorescence intensity profiles from randomly-selected areas of control and 4-AP-stimulated platelets had shown that the surface exposure of CD63 marker on platelet plasma membrane increased 10-fold after stimulation. Therefore, the release of AO exhibited the secretion of platelet secretory granules, particularly, dense bodies and occurred simultaneously with the exocytotic glutamate release shown in Fig. 4.

### 4-AP provoked increase of [Ca^2+^]_*i*_ in the absence of external Ca^2+^

**Figure 6:**
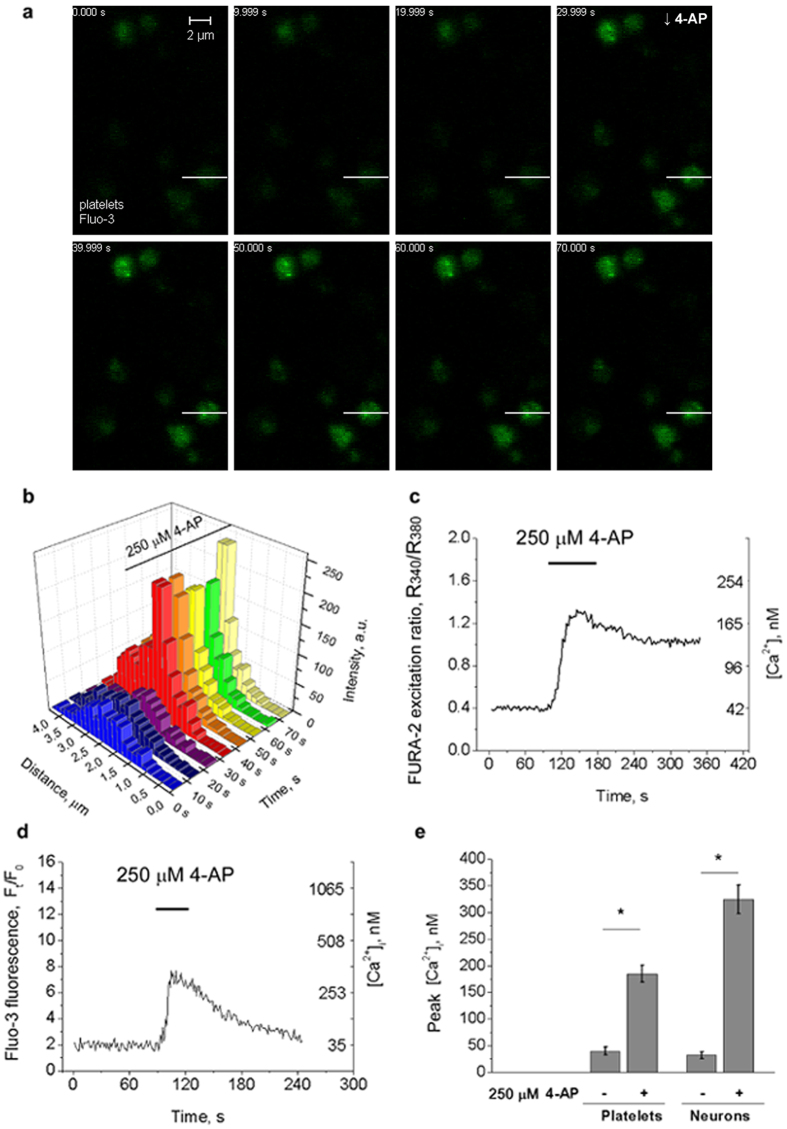
4-AP-induced increase of [Ca^2+^]*_i_* in platelets and neurons in Ca^2+^-free conditions. (**a**,**b)** Confocal imaging of Fluo-3-loaded platelets during the application of 4-AP: **(a)** Typical time series of 4-AP-stimulated increase of [Ca^2+^]_*i*_ in platelets. 4-AP (250 μM) was applied at 30 s. Scale bar is 2 μm; (**b)** The profiles of the fluorescence intensity of Fluo-3-loaded platelet before and after the application of 4-AP. Fluorescence intensity was recorded along white line in (**a**). Profiles are typical for 7 independent experiments; (**c**,**d)** Typical traces of [Ca^2+^]_*i*_ changes in platelets (**c**) and primary neurons (**d**) induced by 4-AP in the absence of external Ca^2+^. 4-AP was applied for the duration indicated by the horizontal bars. Right *y* axis shows the changes in calculated [Ca^2+^]_*i*_, left *y* axis in **c** shows the 340/380 excitation ratio of FURA-2, left *y* axis in (**d**) shows the normalized intensity of Fluo-3 (F_t_/F_0_), the fluorescence normalized to the baseline values after background subtraction; (**e**) The changes of the peak Ca^2+^ concentration in platelets and neurons estimated from 8 independent measurements (*p < 0.05).

Although the experiments were carried out in the absence of extracellular Ca^2+^ the secretory process still required the Ca^2+^ signal. Assuming that the alternative source of Ca^2+^ may originate from intracellular compartments it was important to estimate the [Ca^2+^]_*i*_ in platelets and neurons during 4-AP application. Hence, the cells were loaded with Ca^2+^-sensitive fluorescent dyes: FURA-2, dual excitation ratiometric probe, and Fluo-3 with substantially higher quantum yield. Platelets loaded with Fluo-3/AM were also used for live-cell imaging and the time series were captured before and after 4-AP application as shown in Fig. 6a.

The typical fluorescence intensity profiles of Fluo-3 in Fig. 6b demonstrate the increase (qualitative changes) of [Ca^2+^]_*i*_ in randomly selected platelet within the series (profiles along white line indicated in Fig. 6a). The series of control experiments were performed to confirm that changes of fluorescence were restricted to 4-AP application, while 4-AP itself or possible changes of pH did not influence the emission spectra of acridine orange, Fluo-3, FM1-43 and the excitation spectrum of FURA-2.

It was next important to estimate the changes of the absolute [Ca^2+^]_*i*_ and the relevance of these changes to the stimulation of exocytosis. Quantitative analysis of the changes in [Ca^2+^]_*i*_ with ratiometric probe FURA-2 allows to exclude all unspecific interactions and influences on fluorescent signal that could be misinterpreted as changes in [Ca^2+^]_*i*_. The dissociation constants (*K*_*d*_) of Fluo-3 and FURA-2 after *in situ* calibration consisted of 2,700 nM and ~425 nM, respectively, which differed significantly from *K*_*d*_ determined in the solution. 4-AP induced transient (but with rising 4-AP concentration constant) increase of [Ca^2+^]_*i*_ in platelets from basal 40.2 ± 8.3 nM to 185.5 ± 16.1 nM (Fig. 6c,e). Similar results were obtained with Fluo-3-loaded neurons, where [Ca^2+^]_*i*_ increased to 325.2 ± 27.1 nM (Fig. 6d,e).

The registered 4-AP-stimulated release of pH-sensitive dye and endogenous glutamate from secretory granules together with the Fluo-3 fluorescence intensity profile and surface exposure of secretory granule marker CD63 in the absence of external Ca^2+^ allowed to conclude that 4-AP provoked the sequestration of intracellular Ca^2+^, thereby triggering the exocytosis regardless to the presence of Ca^2+^ in extracellular space.

### 4-AP-stimulated Ca^2+^ efflux from endoplasmic reticulum

**Figure 7:**
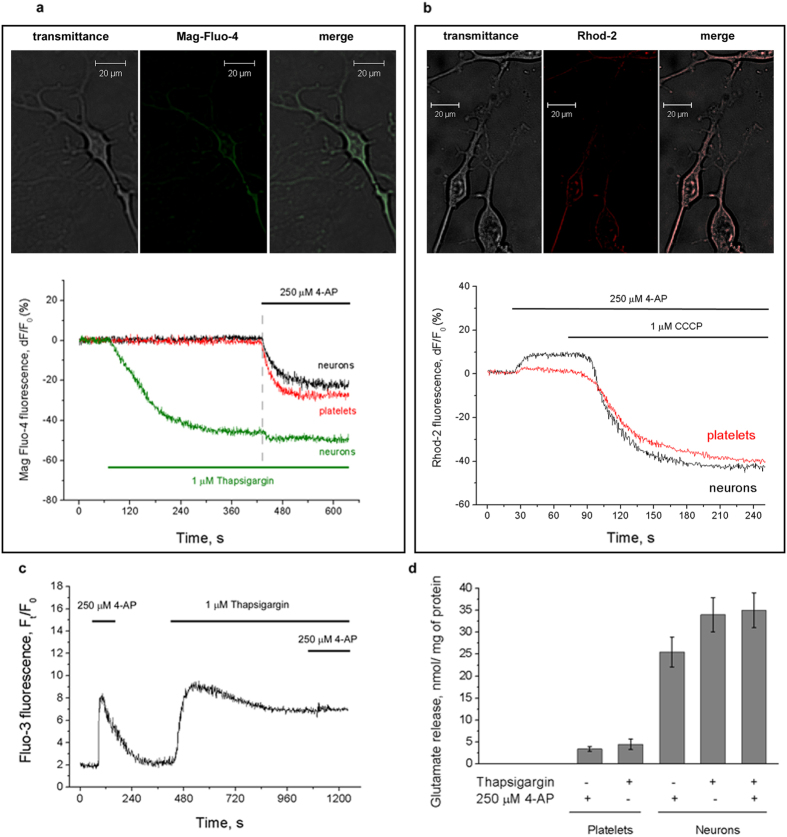
Tracking the changes of [Ca^2+^] in ER and mitochondria of platelets and primary neurons after 4-AP stimulation. Confocal imaging and transmitted light panels (**a**,**b**) demonstrate Mag-Fluo-4 and Rhod-2 localization in primary cortical neurons. (**a)** The profiles of Mag-Fluo-4 fluorescence intensity before and after application of 4-AP (250 μM) and thapsigargin (1 μM). 4-AP (250 μM) was also applied after depletion of Ca^2+^ from ER in neurons (green trace); (**b)** The profiles of Rhod-2 fluorescence in platelets and primary neurons before and after stimulation by 4-AP (250 μM). At the end of recordings the uncoupler CCCP (1 μM) was applied to create the efflux of Ca^2+^ (positive control). Final concentration of platelet/neuronal protein was 0.3 mg/mL; (**c**,**d**) The registration of 4-AP-stimulated [Ca^2+^]_*i*_ rise (**c**) and glutamate release (**d**) before and after the depletion of Ca^2+^ from ER with thapsigargin (1 μM). Endogenous glutamate release was registered using the glutamate dehydrogenase assay. 4-AP, thapsigargin and CCCP (1 μM) were applied for the duration indicated by the horizontal bars. Measurements were made in the absence of external Ca^2+^. Traces are typical for 7 independent experiments.

To determine the intracellular store involved in 4-AP-stimulated [Ca^2+^]_*i*_ rise further analysis was made with fluorescent Ca^2+^-sensitive probes targeted to ER (Mag-Fluo-4)^13^ or mitochondria (Rhod-2)^14^. Mag-Fluo-4 and Rhod-2 localization in primary cortical neurons was demonstrated by confocal imaging (Fig. 7a,b). The changes in the fluorescence intensity were represented as background-subtracted dF/F_0_ (%), where F_0_ was the level of fluorescence before cell stimulation and dF was the change in fluorescence intensity after the application of 4-AP. The monitoring of [Ca^2+^] changes in Mag-Fluo-4-loaded platelets and neurons allowed to demonstrate that 4-AP (250 μM) provoked the sequestration of Ca^2+^ from endoplasmic reticulum (ER) in the absence of external Ca^2+^: the level of Mag-Fluo-4 fluorescence intensity decreased by 23% and 28.5% from initial level in neurons and platelets, correspondingly (Fig. 7a).

There were no detectable changes of mitochondrial [Ca^2+^] found in Rhod-2-loaded platelets (Fig. 7b) after stimulation by 4-AP (250 μM). Interestingly, in primary neurons 4-AP induced additional accumulation of Ca^2+^ in mitochondria (8.5% from basal level) that was abolished by uncoupling agent carbonyl cyanide m-chlorophenyl hydrazone (CCCP).

When ER pool of Ca^2+^ in neurons was depleted with 1 μM thapsigargin, known as irreversible inhibitor of sarcoplasmic/endoplasmic reticulum Ca^2+^-ATPase (SERCA), 4-AP failed to stimulate prominent Ca^2+^ efflux (Fig. 7a).

The registration of 4-AP-stimulated [Ca^2+^]_*i*_ rise and glutamate release before and after the depletion of Ca^2+^ from ER of neurons (Fig. 7c,d) confirmed that changes of [Ca^2+^] in cytosol of stimulated cells and the exocytotic glutamate release were triggered by the sequestration of Ca^2+^ from ER. Platelet activation following thapsigargin application and store depletion made impossible accurate testing of the following 4-AP-stimulated mobilization of Ca^2+^ from ER.

### The chelation of intracellular, rather that extracellular, Ca^2+^ attenuated 4-AP action

**Figure 8:**
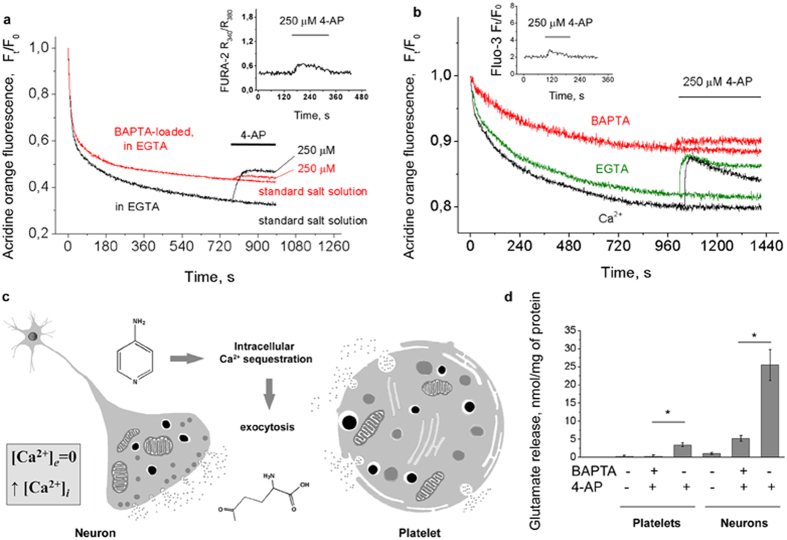
The release of AO and glutamate stimulated by 4-AP after chelation of intracellular Ca^2+^. (**a**,**b)** 4-AP-stimulated release of pH-sensitive dye AO from control and BAPTA-loaded platelets (**a**) and primary neurons (**b**); insets of (**a**,**b**) demonstrate the 4-AP-stimulated [Ca^2+^]_*i*_ rise in BAPTA-loaded preparations. For comparison, see the preparations in Fig. 6c,d; (**c)** Schematic illustration of 4-AP-stimulated glutamate secretion in presynaptic terminal and platelet; (**d)** The release of endogenous glutamate stimulated by 4-AP in control and BAPTA-loaded preparations (*p < 0.05). Final concentration of platelet/neuronal protein was 0.3 mg/mL. Traces are typical for 7 independent experiments.

In order to prove that the mobilization of intracellular Ca^2+^ is the key event in 4-AP action and stimulated glutamate release, the platelets and neurons were loaded with acetoxymethyl ester of intracellular free Ca^2+^ chelator - 1,2-bis(o-aminophenoxy)ethane-N,N,N’,N’-tetraacetic acid (BAPTA). The comparative analysis of 4-AP-stimulated secretion was carried out in the absence of extracellular Ca^2+^ on platelets and neurons (2 mM EGTA), in the presence of 2 mM Ca^2+^ on neurons and on BAPTA-loaded preparations in the absence of extracellular Ca^2+^ (2 mM EGTA, Fig. 8a,b,d). The release of AO from neurons in the absence of extracellular Ca^2+^ (2 mM EGTA) was 12% lower than that in the presence of 2 mM Ca^2+^ (Fig. 8b). This reflects the difference in the dye loading as shown for synaptosomes (Fig. 2c). The following release of the AO from neurons in the absence (EGTA) or presence of Ca^2+^ was subequal, whereas in BAPTA-loaded platelets and neurons the 4-AP-stimulated secretion in Ca^2+^-free medium decreased in average by 80.3 ± 7.2% and 68.2 ± 4.5%, respectively (Fig. 8a,b).

Taking into the account that 250 μM 4-AP did not depolarize plasma membrane (Fig. 2a) the influx of Ca^2+^ from extracellular space through VGCCs could not contribute to the stimulated secretion in the presence of extracellular Ca^2+^.

Considering that the secretion of glutamate is the direct result of Ca^2+^-triggered platelet degranulation or fusion of synaptic vesicles with the presynaptic plasma membrane (Fig. 8c), the comparison was made between the release of endogenous glutamate from control and BAPTA-loaded cells. After loading with BAPTA the release of endogenous glutamate from platelets was at basal (unstimulated) level and decreased by 80% in neurons (Fig. 8d), which may be explained by inability of 4-AP to create sufficient [Ca^2+^]_*i*_ increase in these conditions (Fig. 8a,b, insets). The efficacy of BAPTA loading was controlled by the inhibition of platelet activation and decrease in exocytotic glutamate release from platelets and neurons.

## Discussion

The effect of 4-AP on synaptic transmission is not restricted to the block of voltage-gated potassium channels and plasma membrane depolarization. Known mechanism of 4-AP action implies the presence of external Ca^2+^ and its influx into the cell^4^,^15^. The principal finding of this paper is the ability of 4-AP to stimulate the secretion in platelets, isolated brain nerve terminals and neurons when external Ca^2+^ is depleted. The release of endogenous glutamate from platelets stimulated by 4-AP has been demonstrated for the first time, whereas the more common model for this type of research are primary neuronal cultures, neuromuscular junctions and to lesser extent the neurosecretory cells.

In Ca^2+^-free medium 4-AP was able to stimulate the exocytotic release of synaptic vesicle constituents and platelet dense granules at concentrations below that required to depolarize plasma membrane (50–100 μM). In primary cultured neurons and isolated nerve terminals 4-AP-induced increase in [Ca^2+^]_*i*_ and endogenous glutamate release were observed regardless of the presence of Ca^2+^ in the medium. The external Ca^2+^-independent equal/subequal release of synaptic vesicle constituents (Figs 2c and 4c) suggests that the increase in [Ca^2+^]_*i*_ is not ultimately the result of depolarization-induced Ca^2+^ influx from extracellular space but rather reflects the existence of additional mechanism.

The wider spectrum of 4-AP sensitive objects including platelets, non-excitable cell type, gives rise to the revision of proposed conservative mechanism of 4-AP-stimulated exocytosis. Platelets carry the active transport systems for glutamate, dopamine and serotonin and contain the secretable storage pools of these substances^10^,^16^,^17^,^18^. Glutamate released after 4-AP stimulation (Fig. 4a–c) may originate from secretory pool of glutamate (synaptic vesicles and platelet dense granules) and cytosolic pool because 4-AP promotes the reverse mode of glutamate transporters as a depolarizing agent. Weak depolarization of plasma membrane by 250 μM 4-AP failed to cause stable changes in membrane potential (Fig. 2a), but transporter-mediated glutamate release facilitated during plasma membrane depolarization at more appreciable 4-AP concentrations, may partially contribute to the total release of endogenous glutamate. This is due to the reversibility of glutamate transport, its electrogenicity and high sensitivity to the changes of transmembrane electrochemical gradients^19^,^20^.

At high concentrations (1-2 mM) 4-AP was unable to stimulate the release of AO and endogenous glutamate from isolated synaptic vesicles (Fig. 3), indicating the involvement of another intracellular compartment in mediating the exocytotic response instead of synaptic vesicles. This finding also proves that 4-AP did not affect the spectral properties or redistribution of acridine multimers across the membrane.

In the absence of extracellular Ca^2+^ 4-AP-stimulated glutamate release was accompanied by the destaining of FM1-43-loaded synaptic vesicles (Fig. 2b), providing evidence in favour of the exocytotic process. Moreover, the transporter-mediated release of glutamate is not inherent for platelets and exocytosis is predominant way of glutamate release from platelets^21^. Taken together with the profiles from FM1-43-loaded and 4-AP-stimulated neurons these data support the idea that 4-AP is able to stimulate the secretory process in the absence of external Ca^2+^. In order to confirm or refute this hypothesis, platelets were visualized with fluorescent-labelled antibodies to CD63–a marker of platelet dense secretory granules. As shown in Fig. 5, the surface exposure of dense granule marker CD63 on 4-AP-stimulated platelets increased 10-fold. Since 4-AP induced the degranulation of platelets and increased the surface exposure of CD63 one may expect that 4-AP induces the activation of platelets. However, this activation results from the increase of [Ca^2+^]_*i*_ in platelets and does not involve the initial step of the activation of purinergic and/or PAR receptors as it takes place during clot formation *in vivo*.

The data obtained allowed estimating the endogenous glutamate concentration in dense secretory granules. Considering the amount of dense granules per platelet (3–10), their size (~150 nm diameter), the measured platelet count per mg of protein and the established release of endogenous glutamate from platelets during the stimulation (Fig. 4a) the concentration of glutamate inside the dense granules was approximately 400 μM.

The most important finding is the ability of 4-AP to stimulated [Ca^2+^]_***i***_ rise in neurons and platelets in the absence of extracellular Ca^2+^. The absolute [Ca^2+^]_***i***_ measured with Ca^2+^ sensitive probes (Fig. 6) increased to the level that may trigger the exocytosis^22^. Fluorescent calcium indicators allow to estimate the changes of absolute [Ca^2+^]_*i*_ in nM (Fig. 6). However, Ca^2+^ signal has its temporal characteristics and spatial gradients associated with the movement of Ca^2+^ within neuronal cytoplasm (localized Ca^2+^ spikes and propagating Ca^2+^ waves)^23^,^24^. Localized Ca^2+^ signalling depends on the proximity of the Ca^2+^ sensor and the Ca^2+^ channel. The Ca^2+^ influx through a single Ca^2+^ channel (nanodomain) requires the location of the Ca^2+^ sensor in close proximity to the channel (within 50 nm) for efficient signalling, whereas the summation of Ca^2+^ influxes from clusters of Ca^2+^ channels (microdomain) can trigger exocytosis in active zones within 200 nm^25^.

Intracellular Ca^2+^ chelator, BAPTA, capable of rapid binding with Ca^2+^ and effective buffering of all sorts of Ca^2+^ signals was applied to confirm the direct coupling between the 4-AP-induced Ca^2+^ sequestration and endogenous glutamate release (Fig. 8). In the absence of extracellular Ca^2+^ (2 mM EGTA) the accumulation of AO in BAPTA-loaded cells was lower (Fig. 8a,b) indicating the requirement of cytosolic free Ca^2+^/Ca^2+^ influx for the loading of the dye into additional endosomal pool. Experiments revealed that chelation of extracellular Ca^2+^ with EGTA did not interfere with 4-AP action. In contrast, 4-AP-stimulated release of pH-sensitive dye AO and endogenous glutamate was attenuated in BAPTA-loaded platelets and neurons (Fig. 8a,b). This suggests a profound role of sufficient [Ca^2+^]_*i*_ increase in triggering the exocytotic release of endogenous glutamate.

It should be noted, that at higher concentrations (1-2 mM) 4-AP stimulates 1–the influx of Ca^2+^ from extracellular space through VGCCs as a result of membrane depolarization and 2–the sequestration of Ca^2+^ from ER that is independent of external Ca^2+^ but could be amplified via Ca^2+^-induced Ca^2+^ release mechanism involving ryanodine receptor.

Platelet plasma membrane forms multiple invaginations into the cytoplasm and creates a network of the channels (open canalicular system). This network inside of platelet is in vicinity of the dense tubular system (platelet endoplasmic reticulum, ER)^26^ that represents one of the platelet intracellular Ca^2+^ stores. This enables fast cooperation between the initial stimulus, the generation of second messengers and the release reactions at growing plasmalemmal surface during the pseudopodia formation. The plasma membrane and ER dimension, inherent for neurons and platelets, is essential for cooperation in regulation of the Ca^2+^-dependent processes within the microdomains^27^.

ER has also tight functional coupling and physical proximity to mitochondria that accumulate Ca^2+^ via membrane potential-driven uptake. Agonist-stimulated elevations of [Ca^2+^]_*i*_ evoke rapid and transient increase of mitochondrial [Ca^2+^], which can be prevented by pretreatment with a mitochondrial uncoupler^28^. Fluorescent Ca^2+^-sensitive probes targeted to ER (Mag-Fluo-4) or mitochondria (Rhod-2) enabled the determination of the Ca^2+^ store responsible for 4-AP-stimulated Ca^2+^ efflux. In neurons and platelets 4-AP caused the decrease of [Ca^2+^] in ER similarly to that induced by thapsigargin application (Fig. 7a). No significant contribution of mitochondria in 4-AP-stimulated [Ca^2+^]_*i*_ rise in platelets was found (Fig. 7b). Moreover, previous data suggest that the potential of mitochondrial inner membrane in platelets plays minor role in Ca^2+^ storage or might have an impact at a higher cytosolic Ca^2+^ concentration^29^.

In contrast to platelets, 4-AP stimulation of neurons increased mitochondrial [Ca^2+^] that suggests the involvement of mitochondrial Ca^2+^ uptake in termination of Ca^2+^ signal. This is consistent with the finding that upon opening of the inositol 1,4,5-triphosphate-gated channels of the ER, the mitochondrial surface is exposed to a higher concentration of Ca^2+^ as compared to bulk cytosol. This emphasizes the importance of cell architecture and the distribution of organelles in compartmentalization and regulation of Ca^2+^ signalling^30^.

The analysis of [Ca^2+^]_*i*_ rise and endogenous glutamate release before and after the depletion of Ca^2+^ from ER (Fig. 7c,d) demonstrated that the sequestration of Ca^2+^ and 4-AP-induced glutamate release proceeded in the absence of extracellular Ca^2+^ and were abolished by pretreatment with thapsigargin. This further proves that 4-AP triggered the exocytosis by the sequestration of Ca^2+^ from ER.

The efficiency of 4-AP had been proven at the medical treatment of Lambert–Eaton myasthenic syndrome^31^. This disorder is characterized by muscle weakness of the limbs resulting from the production of antibodies against presynaptic voltage-gated calcium channels (VGCCs) and, possibly, some other proteins of neuromuscular junction^32^. It is worth of noticing that under these conditions the additional mechanism of Ca^2+^ mobilization, independent of VGCCs, may retain physiological [Ca^2+^]_*i*_ rise much more efficiently. The sequestration of Ca^2+^ via VGCCs stimulated by direct depolarization of adjacent membrane takes place at the neuromuscular junctions. The analogous cascade may also be of value for platelets. The depolarization of the platelet plasma membrane may propagate among the OCS provoking modulation of VGCCs and the adjoining membrane of dense tubular system.

Increase in [Ca^2+^]_*i*_ affects wide range of cellular signalling pathways, vesicle trafficking and makes a strong impact on the observed processes, particularly, on transport of neurotransmitters at the level of plasma membrane and synaptic vesicles. These results require careful consideration in future experimental work, since the ability of 4-AP to induce depolarization of plasma membrane and neurotransmitter release is commonly used in neurophysiological and neurochemical research.

## Conclusions

The results presented in this work demonstrate that Ca^2+^-sequestration is an important component in the mechanism of 4-AP action on different types of target cells and unveil the role 4-AP plays as a potentiator of synaptic and neuromuscular transmission. 4-AP combines the properties of depolarizing agent and the ability to sequester Ca^2+^ from intracellular compartments. This complex action underlies the efficiency of 4-aminopyridine as an active substance of drugs for managing of multiple sclerosis and conditions related to reduced Ca^2+^ efflux from intracellular stores.

### Materials

L-glutamic acid, 4-aminopyridine, glutamate dehydrogenase (G2626, EC 1.4.1.3), NaCl, KC1, MgCl_2_, NaH_2_PO_4_, EGTA, EDTA, HEPES, D-glucose, NAD^+^, thapsigargin, CCCP, anti-CD63-mAbs-APC (SAB4700216), minimum essential medium, FBS, poly-D-lysine, A23187 and digitonin were purchased from Sigma-Aldrich (USA), Ficoll-400–from Amersham (UK). Acridine orange, rhodamine 6G, FM1-43, Fluo-3/AM, FURA-2/AM, Mag-Fluo-4/AM and Rhod-2/AM were obtained from Molecular Probes (USA). All chemicals used were of analytical grade. The water solutions were made with deionised water.

### Ethics statement

Animal care, blood sampling and euthanasia, were carried out in accordance with the European Guidelines and International Laws and Policies and were approved by the Institutional Animal Care and Use Committee, Palladin Institute of Biochemistry, NAS of Ukraine (protocol N2 from 12.04.2014).

### Isolation of brain nerve terminals (synaptosomes)

The male rats were sacrificed by decapitation. Cerebral hemispheres were removed and homogenized in ice-cold 0.32 M sucrose, 5 mM HEPES–NaOH (pH 7.4) and 0.2 mM EDTA. Synaptosomes were prepared by differential and Ficoll-400 density gradient centrifugation of rat brain homogenate at 4 °C as described by Cotman^33^. The pellet of isolated nerve terminals was resuspended in oxygenated HEPES-buffered Krebs-Ringer solution. Free Ca^2+^ concentrations were set with Ca^2+^/EGTA buffers.

### Isolation of synaptic vesicles

Synaptic vesicles were isolated from nerve terminals of brain cerebral hemispheres using the hypo-osmotic shock (1 mM EGTA, 10 mM Tris-HCl, pH 8.1) at 4 °C for 60 min. Synaptic membranes were isolated by centrifugation at 20,000 g for 30 min. After centrifugation of cytosolic fraction at 55,000 g for 60 min the supernatant was used to obtain the pellet of synaptic vesicles (130,000 g, 4 °C for 60 min)^34^.

### Primary neuronal cultures

Micro-dissected cortices from one-day-old mouse pups were incubated with trypsin and DNAse I for 15 minutes at 37 °C. Before mechanical dissociation, cells were washed thoroughly and incubated for 15 min in inactivating solution (trypsin inhibitor and 10% FBS in minimum essential medium). Obtained mass was triturated and passed through 40-μm nylon mesh cell strainer. Single cell suspension was washed and resuspended in plating medium. Cell count and viability were determined using haemocytometer and trypan blue exclusion assay.

Glial feeder cultures were prepared in advance from cortices of the newborn mouse pups using the trypsinization, adherence-based selection of astrocytes and generation of monolayers with approx 60% confluence. For co-culturing, primary neurons were plated (1 × 10^6^ cell/mL) on poly-D-lysine-precoated 6-well plates. The preconditioned astrocyte feeder cultures for trophic support were seeded on porous polyester membrane of Transwell inserts with 0.4 μm pore size. Experiments were carried out after 14 days of co-culturing.

### Isolation of blood platelets

Blood samples were collected from male rabbits and rapidly centrifuged at 400 g for 10 min to obtain platelet-rich plasma. The upper liquid phase above the white blood cell interface was carefully remove and centrifuged at 3,000 g for 10 min. The pellet was re-suspended in standard Krebs-Ringer solution (pH 7.4). Platelets were used in the experiments for up to 3 hours after blood sampling without loss of response to activators (2 μM ADP, 0.1 NIH U/mL thrombin).

To exclude the activated platelet preparations based on dot plots of side scatter versus forward scatter and to evaluate the cell count the flow cytometry analysis was performed using COULTER EPICS XL flow cytometer (Beckman Coulter, USA). For each sample 20,000 events were acquired.

### Monitoring of FM 1-43 destaining in neurons

Synaptic vesicle recycling in primary cultured neurons was registered with fluorescent styryl dye FM1-43 that reversibly binds to the neuronal plasma membrane and becomes trapped within endosomes/recycled synaptic vesicles during endocytosis. To stimulate vesicle turnover and dye uptake the isosmotic substitution of Na^+^ for K^+^ was performed resulting in final K^+^ concentration of 70 mM. Synaptic vesicles were allowed to take up the dye (5 μM) for 20 min before being washed twice. Before the initiation of destaining the steady-state level of fluorescence was registered to exclude the spontaneous exocytosis. Destaining was calculated in percentage of total accumulated dye.

### Measurements of [Ca^2+^]_*i*_

Cells were loaded with ratiometric indicator FURA-2/AM (1 μM) or Fluo-3/AM (4 μM) at 30 °C for 60 min. BAPTA/AM loading started 30 min after FURA-2/AM loading. Then, cells were washed and left for 30 min to let complete de-esterification. Measurements were performed using the QuantaMaster 40 spectrofluorimeter (Photon Technology International, Inc., Canada).

In dual-excitation mode FURA-2-loaded samples were excited alternately at 340 and 380 nm and fluorescence was recorded at 510 nm to calculate the 340/380 excitation ratio. Fluo-3-loaded samples were excited at 506 nm and the fluorescence intensity changes were recorded at 526 nm. The intensity of Fluo-3 was normalized to baseline after background subtraction (F_t_/F_0_). Background values were obtained after permeabilization of the cells with digitonin and washout of Fluo-3 at the end of experiments. *In situ* calibrations were conducted in the presence of 5 μM A23187. The intracellular [Ca^2+^] was calculated according to the Grynkiewicz, Poenie and Tsien equation^35^.

For measurements of [Ca^2+^] in intracellular stores primary cultured neurons and platelets were loaded with Rhod-2/AM (4 μM) or Mag-Fluo-4/AM (5 μM) for 1 h at 30 °C and then left to de-esterify in dye-free medium for 2 h. Rhod-2-loaded samples were excited at 530 nm and the fluorescence intensity was recorded at 580 nm. Mag-Fluo-4-loaded samples were excited at 480 nm and the fluorescence intensity was recorded at 520 nm. The intensities were normalized to baseline after background subtraction.

### Laser scanning confocal imaging

For confocal imaging platelets were loaded with Fluo-3/AM, Rhod-2/AM and Mag-Fluo-4/AM as described in previous section. The staining with anti-CD63-APC was carried out at 4 °C for 30 min. Platelets were then washed, plated on slides and viewed under the confocal laser scanning microscope LSM 510 META, Carl Zeiss, with objective Plan-Apochromat 100 × /1.4 Oil DIC at 37 °C. For registration of the changes in [Ca^2+^]_*i*_ in Fluo-3-loaded platelets the specimens were excited at 488 nm and emission was collected through a 505-nm long pass filter.

The specimens with anti-CD63-APC-labeled platelets were excited with 633-nm laser and emission was collected through a 650-nm long pass filter. Cortical neurons loaded with Rhod-2/AM were excited with 543-nm laser and emission was collected using a 560–615 nm band-pass filter. Mag-Fluo-4-loaded cells were excited at 488 nm and emission was collected using a 505–530 nm band-pass filter.

Images and time series were captured with high-resolution digital camera AxioCam HRc. Acquisition software: Zeiss LSM Image Browser Version 4.0.0.241.

### Measurements of the acidification of intracellular compartments

The changes in the acidification of intracellular compartments were registered with pH-sensitive fluorescent dye acridine orange (AO) that tends to form dimers and multimers after protonation at acidic pH. This results in decrease of green fluorescence that is inherent for monomeric dye. Platelets/synaptosomes/isolated synaptic vesicles or neurons were preincubated at 30 °C for 10 min. Fluorescence intensity measurements were started with application of AO (5 μM) and recorded at excitation and emission wavelengths of 490 and 530 nm, respectively. The 4-AP was applied at the steady-state level of dye accumulation. The curves were normalized to the similar traces in the absence of cells/synaptic vesicles according to Equation 1:


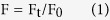


where *F*_*t*_ and *F*_*0*_ are the fluorescence intensities of acridine orange with and without cells/synaptic vesicles, respectively.

### Monitoring changes in membrane potential of primary cortical neurons

The changes of plasma membrane potential were measured using the potentiometric fluorescent dye rhodamine 6G. Cortical neurons were preincubated at 30 °C for 10 min and the measurements were started by application of rhodamine 6G at a final concentration of 0.5 mM. Fluorescence intensity was recorded at excitation and emission wavelengths of 528 and 551 nm, respectively. The 4-AP was applied at the steady-state level of dye accumulation. To estimate the changes of membrane potential the curves were normalized to the similar traces in the absence of cells according to Equation 1, where *F*_*t*_ and *F*_*0*_ are the fluorescence intensities of rhodamine 6G with and without cells, respectively.

### Glutamate dehydrogenase assay

The release of endogenous glutamate was registered using the glutamate dehydrogenase assay^36^,^37^ based on formation of reduced β-nicotinamide adenine dinucleotide (NADH) with fluorescent peak at 460 nm.

Platelets/synaptosomes/neurons/synaptic vesicles (final protein concentration of 0.5 mg/mL) were incubated at 30 °C for 10 min with glutamate dehydrogenase (20 U/mL) and NAD^+^ (1 mM) in cuvette under continuous stirring condition. Endogenous glutamate release was registered with excitation at 340 nm and emission at 460 nm. At the end of measurements different concentrations of exogenous glutamate were applied for calibration of the fluorescent signal.

### Statistical analysis

Data are presented as means with standard error of the mean (s.e.m.) for *n* independent experiments. The significant difference between the means was detected by one-way ANOVA at the 0.05 significance level. The absence of prominent exocytotic peak (25–30% from accumulated dye) typical for functional synaptosomes and neurones (insets of Fig. 1b,d, respectively) was the criterion for data exclusion.

## Additional Information

**How to cite this article**: Kasatkina, L. A. 4-Aminopyridine sequesters intracellular Ca^2+^ which triggers exocytosis in excitable and non-excitable cells. *Sci. Rep.* **6**, 34749; doi: 10.1038/srep34749 (2016).
